# The Role of Transjugular Intrahepatic Portosystemic Shunt in Management of Peribiliary Varices

**DOI:** 10.14309/crj.0000000000001839

**Published:** 2025-09-25

**Authors:** Wael Mohamed, Mohamed Omar, David Maundu, Kyle Rowe

**Affiliations:** 1 Department of Gastroenterology, University of Kansas School of Medicine-Wichita, Wichita, KS; 2 Department of Internal Medicine/Pediatrics, University of Kansas School of Medicine-Wichita, Wichita, KS

**Keywords:** transjugular intrahepatic portosystemic shunt, peribiliary varices, portal hypertension, cirrhosis, variceal bleeding, endovascular intervention, hepatic encephalopathy, portal pressure reduction, variceal embolization, salvage therapy, secondary prophylaxis, hepatology

## Abstract

Peribiliary varices are a rare complication of chronic portal hypertension and can lead to common bile duct narrowing. Because of this, they can complicate the management of biliary obstruction secondary to choledocholithiasis. Instrumentation such as during endoscopic retrograde cholangiopancreatography poses the risk of severe hemorrhage. As such, decompressing the portal circulation via methods such as transjugular intrahepatic portosystemic shunt before attempted biliary stone extraction and sphincterotomy may be necessary to facilitate safe and successful treatment. This case demonstrates the crucial role of transjugular intrahepatic portosystemic shunt placement in enabling successful intervention in a challenging scenario where peribiliary varices obstructed access to the common bile duct.

## INTRODUCTION

Peribiliary varices, also known as choledochal varices, arise from dilation of the venous plexuses that run along the biliary tree. They are relatively rare and have been noted to frequently arise in cases of extraportal hepatic vein obstruction.^1,2^ Most patients with peribiliary varices are asymptomatic. However, since the varices reduce the caliber of the ducts, these patients are predisposed to repeated biliary obstruction either via a stone or stricturing secondary to the varix development. Patients may, therefore, exhibit symptoms such as fever, chills, jaundice, or abdominal pain.^3^ Laboratory findings will typically show elevated bilirubin levels, in particular direct bilirubin, alkaline phosphatase, and gamma-glutamyl transferase.

Because of their location, these varices pose a unique challenge when attempting removal of the biliary stones via endoscopic retrograde cholangiopancreatography (ERCP). Key among these difficulties is the risk of severe intraoperative hemorrhage, infection, and possible mortality.^4^ In a previous systematic review of ERCP procedures performed for portal biliopathy, hemobilia was a recognized complication occurring in 4% of procedures and the transient pressure elevations during balloon sweeping increased the risk of bleeding.^1^ This case demonstrates the use of adjunctive therapies such as transjugular intrahepatic portosystemic shunt (TIPS) in facilitating successful ERCP.

## CASE REPORT

A 63-year-old man with a history of cirrhosis because of metabolic associated fatty liver disease and multiple chronic portal venous occlusion presented with abdominal pain, nausea, dyspnea, fever, and new-onset jaundice. On physical examination, the patient was febrile and had generalized abdominal pain but no guarding or tenderness. Laboratory values showed the following values: total bilirubin was elevated at 6.0 mg/dL (0–1.0); direct bilirubin, 4.8 mg/dL (0–0.3). His liver enzymes were likewise elevated with aspartate aminotransferase (AST), 203 U/L (10–37); alanine aminotransferase (ALT), 194 U/L (<66); alkaline phosphatase 934 IU/L (45–117); and albumin 2.3 g/dL (3.4–5.0). He had leukocytosis at 15,600/mm^3^ (5,000–10,000) but normal platelets at 158 k/mm^3^ (150–400). A chest X-ray done showed basal atelectasis and pulmonary edema. An abdominal computed tomography scan revealed splenomegaly with multifocal splenic infarcts, a cirrhotic liver morphology and intrahepatic and extrahepatic biliary duct dilation. His Model for End-Stage Liver Disease score was 18 and was driven mainly by the high bilirubin.

**Figure 1.**
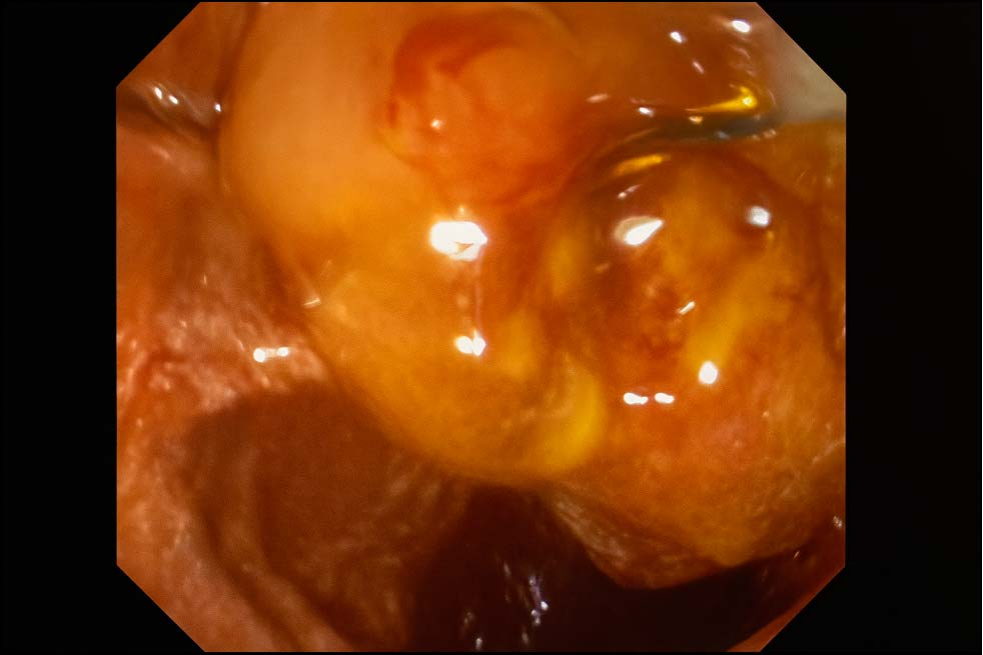
Decompressed peribiliary varices.

**Figure 2.**
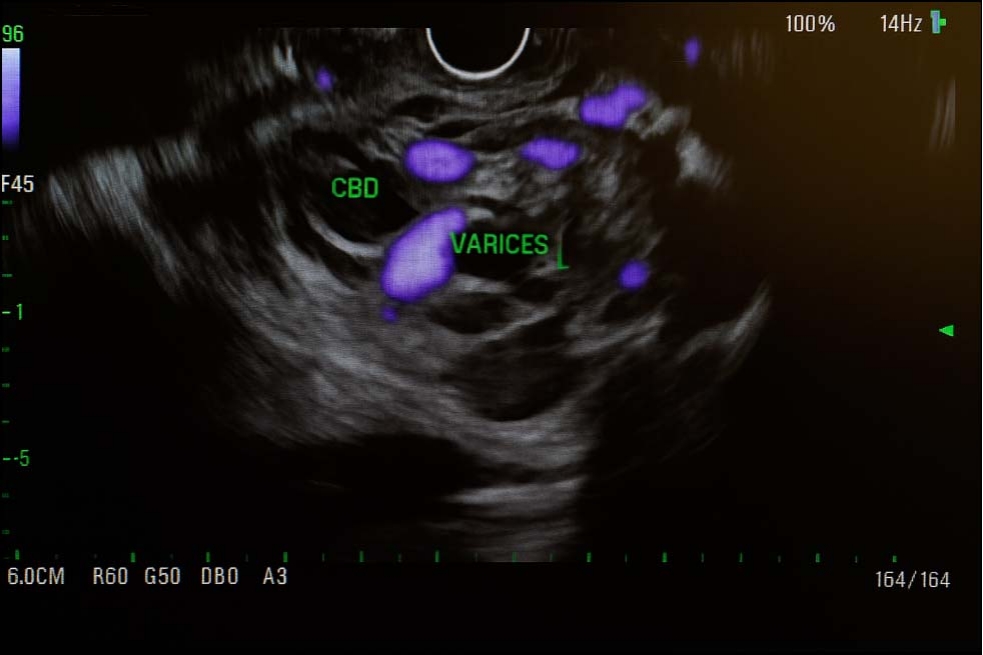
MRCP showing peribiliary varices.

Intrahepatic and common bile dilatation was initially seen on magnetic resonance cholangiopancreatography, and he was initially scheduled for endoscopic ultrasound and ERCP (Figure 2). During endoscopic ultrasound, the patient was found to have grade 2 esophageal varices, gastric varices, and severe portal hypertensive gastropathy. Notably, large intracholedochal peribiliary varices were discovered compressing the common bile duct, with a distal choledocholithiasis noted (Figure 1). In addition, duodenal bulb stenosis was identified because of extrinsic compression from the varices. ERCP was deferred owing to the high risk of severe intraoperative bleeding posed by the large peribiliary varices.

**Figure 3.**
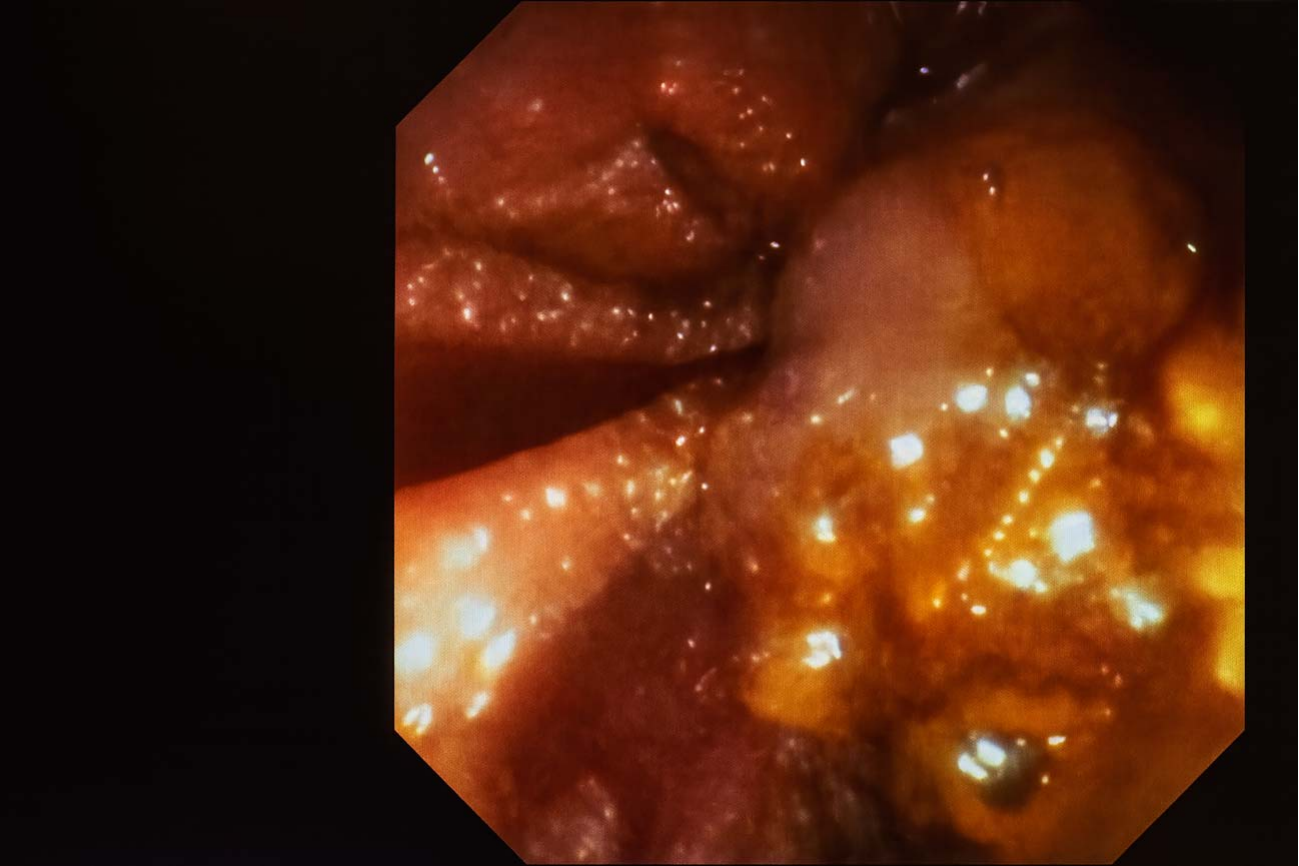
Post TIPS endoscopy showing decompressed duodenal bulb varices.

As a result, the patient was referred to interventional radiology for a TIPS placement to decompress the portal circulation for which access was obtained through the right internal jugular vein. A decision to perform partial particle embolization of the lower pole of the spleen was made as a result of the marked splenomegaly noted. TIPS placement was not contraindicated given the absence of prior hepatic encephalopathy and his Model for End-Stage Liver Disease score. Three days after the TIPS placement, ERCP was performed, which demonstrated significant improvements in the Grade I esophageal, gastric, and most importantly intracholedochal peribiliary varices (Figure 3). The biliary stones were successfully extracted as well as copious sludge drained. A 10-F plastic biliary stent was placed, which showed excellent drainage. No special precautions or modifications were performed during the procedure as the patient was deemed to have a reduced risk of bleeding.

## DISCUSSION

Peribiliary varices are a relatively rare presentation of portal hypertension, which can complicate the management of biliary obstruction. They develop when portal hypertension causes the opening of venous collaterals that drain into the systemic circulation.^5^ Whereas this more commonly leads to development of esophageal and gastric varices, this process can lead to formation of collaterals in the epicholedochal and paracholedochal venous plexuses.^3,6^

These varices lead to alteration of the normal smooth intraluminal surface of the common bile duct. They protrude into the lumen and cause an irregular mural surface, which can promote formation and stasis of any choledocholithiasis.^6^ The bile duct wall is thin and pliable, and these changes increase the risk of bleeding when undergoing instrumentation when trying to extract the stones during ERCP.^7^ Magnetic resonance cholangiopancreatography is a good noninvasive modality used to assess these changes and can be used to make the diagnosis before intervening endoscopically. As in our patient, it is possible to note intrahepatic dilation as well as common bile duct dilation. Filling defects seen can be indicative of cholelithiasis.

In patients for whom endoscopic treatment poses a significant risk of intraoperative bleeding, decompression of the portal circulation is a necessary preceding step.^1,8^ Surgery has traditionally been the treatment of choice in patients with ectopic varices for whom endoscopic therapy fails. However, it is invasive and should be reserved for patient who fail TIPS placement. TIPS is less invasive, and by creating a portocaval shunt, it helps in decompressing the portal circulation and reducing the size of the varices.^7^ A 2017 guideline from the American Association for the Study of Liver Diseases recommended a multidisciplinary approach to treating ectopic varices with TIPS being one of the modalities suggested in managing this condition.^9^ However, the quality of evidence behind this recommendation is low.

In our case, TIPS placement significantly reduced the size of the peribiliary varices, which facilitated easier intervention on repeat ERCP. This illustrates the benefit of using TIPS in such cases.

## DISCLOSURES

Author contributions: W. Mohamed is the lead author and was responsible for conceptualization; investigation; data curation; visualization; writing—original draft. M. Omar and D. Maundu were the second and third authors responsible for investigation; data curation; literature search; writing—review and editing. K. Rowe is responsible for patient evaluation and critical case revisions and is the article guarantor.

Financial disclosure: None to report.

Informed consent was obtained for this case report.

## ABBREVIATIONS:

10-F: ten french
ALT: alanine aminotransferase
AST: aminotransferase
ERCP: endoscopic retrograde cholangiopancreatography
g/dL: grams per deciliter
IU/L: international units per liter
mg/dL: milligrams per deciliteraspartate
TIPS: transjugular intrahepatic portosystemic shunt
U/L: units per liter
